# PP59 Do Patient Contributions Matter? A Thematic Document Analysis Of NICE Ultra-Rare Disease Appraisals

**DOI:** 10.1017/S0266462324002307

**Published:** 2025-01-07

**Authors:** Arianna Gentilini, Alina Rana

## Abstract

**Introduction:**

Patient organizations play a crucial role in health technology assessment (HTA), especially for rare diseases. Despite their important role, the contributions and impact of patient organizations have been overlooked in the literature. This study aims to address this gap by examining the contributions of patient organizations and their nominated experts in National Institute for Health Care and Excellence (NICE) highly specialised technology (HST) appraisals.

**Methods:**

We thematically analyzed the scope and frequency of contributions from patient organizations and experts associated with 10 NICE HST appraisals completed between January 2022 and September 2023. First, to allow for a representation of what patient contributions focus on, their written submissions were categorized according to themes following a deductive/inductive approach, employing a tiered system for disease-, technology-, or submission-specific themes. Second, we compared the themes identified in written submissions with those found in the final NICE recommendations, included in the final evaluation determination (FED), to understand whether and to which extent patients’ contributions were considered.

**Results:**

From 2013 to 2023, 22 drugs underwent HST assessment, with nearly half appraised during 2022 and 2023. All technologies received positive recommendations. A total of 475 unique patient contributions—from both patient organizations and their nominated experts—were identified in their written submission in support of the 10 HST appraisal assessed, predominantly emphasizing disease-specific themes (53%), such as quality of life. While 42 percent of raised themes aligned with FED content, 52 percent did not. When looking at individual appraisals, the share of themes mentioned in patients’ written submission explicitly considered in the FEDs ranged from nine percent to 73 percent, with a median of 50 percent.

**Conclusions:**

Despite progress in integrating patient inputs into HTA, this study reveals a discrepancy between patient priorities and explicit consideration in NICE’s final recommendations. While NICE consistently adheres to its methodology, certain patient-raised aspects are overlooked. Further research is important to discern the optimal areas and timing for patient contribution, refining NICE’s involvement strategies in their decision-making processes.

